# Cell‐free DNA as a biomarker after lung transplantation: A proof‐of‐concept study

**DOI:** 10.1002/iid3.620

**Published:** 2022-04-19

**Authors:** Jesper M. Magnusson, Anne Ricksten, Göran Dellgren, Carina Wasslavik, Rickard Nordén, Johan Westin, Jens Boehmer

**Affiliations:** ^1^ Transplant Institute Sahlgrenska University Hospital Gothenburg Sweden; ^2^ Department of Respiratory Medicine, Institute of Medicine, Sahlgrenska University Hospital University of Gothenburg Gothenburg Sweden; ^3^ Department of Clinical Chemistry Sahlgrenska University Hospital Gothenburg Sweden; ^4^ Department of Clinical Chemistry and Transfusion Medicine, Institute of Biomedicine, Sahlgrenska University Hospital University of Gothenburg Gothenburg Sweden; ^5^ Department of Cardiothoracic Surgery, Institute of Medicine, Sahlgrenska University Hospital University of Gothenburg Gothenburg Sweden; ^6^ Department of Clinical Microbiology Sahlgrenska University Hospital, Region Västra Götaland Gothenburg Sweden; ^7^ Department of Infectious Diseases, Institute of Biomedicine, Sahlgrenska University Hospital University of Gothenburg Gothenburg Sweden; ^8^ Department of Pediatrics, Queen Silvias Children's Hospital Sahlgrenska University Hospital Gothenburg Sweden; ^9^ Department of Cardiology, Institute of Medicine, Sahlgrenska University Hospital University of Gothenburg Gothenburg Sweden

**Keywords:** biomarker, BOS, lung injury, native/allograft dysfunction

## Abstract

**Background:**

Lung transplantation (LTx) is a lifesaving procedure burdened with limited long‐term survival. The most common cause of death after LTx is chronic lung allograft dysfunction (CLAD). Today, useful biomarkers for the detection of CLAD are lacking. Circulating cell‐free DNA (cfDNA) is released during cellular decay and can be detected using polymerase chain reaction (PCR). Thus, donor‐derived cfDNA in recipient serum indicates cellular decay in the transplanted organ. In the current study, we explore the possibility of using a novel PCR method to detect cfDNA as a biomarker for clinical events, especially CLAD.

**Methods:**

Four patients were retrospectively tested for levels of both donor and recipient‐derived cfDNA using digital droplet PCR after targeted preamplification. The results were correlated to recorded clinical events.

**Results:**

All available samples rendered results. Both patients that later developed CLAD showed a persistently elevated ratio between donor‐and recipient‐derived cfDNA. Also, the mean level of cfDNA was higher in the two patients who later developed CLAD than in patients who did not (*p* = .0015).

**Conclusions:**

This proof‐of‐concept study suggests that cfDNA quantified with PCR may be used as a biomarker of significant clinical events such as CLAD.

## Graphical abstract

Droplet digital polymerase chain reaction‐detected serum cell‐free DNA (cfDNA) covaries with chronic lung allograft dysfunction (CLAD) and infectious severe events in a proof‐of‐concept study of a novel method for biomonitoring of lung transplant recipients.

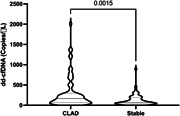

## 1 INTRODUCTION

In irreversible nonmalignant lung disease, lung transplantation (LTx) can be the sole option for lifesaving treatment. Almost 70,000 lung transplants have been performed internationally until today,^1^ and more than 45 procedures are currently performed annually at our transplant centre.^2^ However, LTx is still burdened by limited long‐term survival.^3^ The main limiting factor is the development of bronchiolitis obliterans syndrome (BOS).^4^ BOS represents a subset of chronic lung allograft dysfunction (CLAD).^5^ CLAD is defined by an irreversible loss of 20% or more of a baseline forced expiratory volume one second (FEV1) when other causes have been excluded. Thus, irreversible damage to the lung is already present at the time of diagnosis. Several risk factors for CLAD development have been identified, such as primary graft dysfunction,^6^ viral infections,^7^, ^8^ esophageal reflux,^9^ as well as acute cellular rejection.^10^ However, the occurrence of risk factors does not adequately predict CLAD development, and there is currently insufficient understanding of the mechanisms underlying allograft damage. No functional biomarker has proven beneficial in diagnosing clinical events, especially CLAD, after lung transplantation. A valuable biomarker for allograft damage would facilitate early detection of CLAD in a clinical setting and thus enable early therapeutic intervention,^11^ which would be an opportunity to improve outcomes after LTx.

Cell‐free DNA (cfDNA) is released from cells into the surrounding tissue and bloodstream during apoptotic and necrotic cellular decay and can be detected in plasma and serum.^12^ An association exists between circulating levels of cfDNA and disease severity of traumatic injuries,^13^ sepsis,^14^ and malignant disease.^15^ Two distinct DNA sets exist in one individual (from the donor and the recipient), following all organ transplantations. Improved technical development has made it possible to differentiate donor‐derived cfDNA (dd‐cfDNA) from recipient‐derived cfDNA (rd‐cfDNA) in the bloodstream.^16^ Thus, quantification of each portion can be made, and the ratio of dd‐cfDNA to all cfDNA, also called the donor fraction (DF), has been associated with graft injury after kidney,^17^ liver,^18^ heart^19^ and lung^20^ transplantation. A method using targeted preamplification and droplet digital polymerase chain reaction (ddPCR) to quantify dd‐cfDNA and rd‐cfDNA levels have been developed.^21^, ^22^ The possibility to report on both donor and recipient levels of cfDNA provides an improved possibility for interpretation compared to previously published methods using sequencing techniques with DF as the sole‐reported variable.^20^, ^23^, ^24^ Furthermore, a recent review, in addition to previous points, also highlights the potential cost‐effectiveness of reporting each fraction separately.^25^ We applied the current method on previously stored samples from a prospective surveillance study, with a confirmed high degree of clinical events coverage.^8^

To evaluate the potential usefulness of the method for lung transplant recipients, this pilot study aimed to in a limited, select population, explore the potential association between levels of PCR‐detected cfDNA in serum and the timing and severity of various clinical events, with a particular emphasis on CLAD after lung transplantation.

## 2 MATERIALS AND METHODS

### 2.1 Patients and study design

Ninety‐eight patients who underwent LTx between 2009 and 2011 at Sahlgrenska University Hospital were prospectively included in a follow‐up study designed to investigate the impact of infectious complications on the outcome following LTx. Data from this study has been the subject of previous publications.^8^, ^26^ Serum samples were collected at scheduled outpatient visits after LTx at 1, 2, 3, 4.5, 6, 9, 12, 18, 24, and 36 months. Furthermore, samples were also collected at every extra outpatient visit during this time period. All extra visits were prompted by respiratory symptoms and occurred at the transplant unit. All serum samples were centrifuged at 3000*g* and aliquoted before they were frozen at −80°C within 24 h after sampling. Surveillance of infectious complications was performed with a multiplex real‐time PCR assay as previously described^27^ for viral infections and regular cultures for bacterial and fungal infections. Symptoms of infectious complications, acute rejections, and CLAD were recorded in an electronic case report form. After the end of the follow‐up, all patients were reviewed by two experienced transplant pulmonologists in a blinded fashion for CLAD diagnosis. Additional clinical data were retrieved from electronic patient charts.

Four patients were selected for this exploratory proof‐of‐concept investigation. Eligible patients were patients without retransplantation, with not more than three recorded clinical events and at least five sequential serum samples available. Patients with variable FEV1 and CLAD at end of follow‐up were identified from this group. Patients without CLAD and intermittent FEV1 loss during follow‐up were identified as controls. From each of these two groups, two patients were selected at random. For the current study, both CLAD diagnoses were reevaluated to be adherent to the 2019 definition.^5^ The project was approved by the ethical review board in Gothenburg (Dnr: 791‐08). All participants provided written informed consent.

The laboratory staff was blinded to all clinical and patient‐related data. Serum samples were identified by serial numbers only during analysis and data management.

Base immunosuppression protocol for each patient is outlined in Table 1 and has been previously described in detail.^27^ Airway infections prompted a transient 1‐to‐3‐week elevation of prednisone to approximately 0.3 mg/kg. No other adjustments to base immunosuppression were made based on clinical events for any of the patients.

**Table 1:** Patient characteristics and clinical events

*Patient 1*					
Age	Sex	Diagnosis	Transplant type	CMV mismatch	Immunosuppression
53	Male	IPF	Double lung	No	Ciclosporin, MMF, prednisone
Clinical event 1 at 4.5 months		Corona OC43 infection with airway symptoms			
Clinical event 2 at 16 months		CMV reactivation with viraemia			
CLAD at 30 months					
*Patient 2*					
Age	Sex	Diagnosis	Transplant type	CMV mismatch	Immunosuppression
62	Female	COPD	Double lung	No	Ciclosporin, MMF, prednisone
Clinical event 1 at 6 months		Cutaneous herpes zoster infection			
CLAD at 16 months					
*Patient 3*					
Age	Sex	Diagnosis	Transplant type	CMV mismatch	Immunosuppression
63	Male	IPF	Single lung	No	Ciclosporin, MMF, prednisone
Clinical event 1 at 6 months		Native lung infection			
Clinical event 2 at 12 months		Sample after biopsy			
*Patient 4*					
Age	Sex	Diagnosis	Transplant type	CMV mismatch	Immunosuppression
53	Male	Sarcoidosis	Double lung	No	Tacrolimus, MMF, prednisone
Clinical event 1 at 4.5 months		Deterioration, possibly acute cellular rejection			
Clinical event 2 at 18 months		Asymptomatic, rhinovirus infection			

Abbreviations: CLAD, chronic lung allograft dysfunction; CMV, cytomegalovirus; COPD, chronic obstructive pulmonary; diseaseIPF, idiopathic pulmonary fibrosis; MMF, mycophenolate mofetil.

CLAD was defined as an irreversible loss of >20% of baseline FEV1, confirmed with at least two spirometries at least 3 weeks apart, where other differential diagnoses had been excluded. Furthermore, treatment with azithromycin for at least 3 months without any signs of restitution was added as a criterion to be included as definitive CLAD in the current study.

### 2.2 DNA isolation and genotyping

Whole blood samples were used for genotyping. Donor and recipient genomic DNA was extracted from EDTA‐blood preparations using the DNeasy Blood & Tissue Kit (Qiagen).

Serum samples were used for longitudinal detection of cfDNA. cfDNA was extracted from 0.25 to 1.25 ml serum using the QIAamp® Circulating Nucleic Acid Kit (Qiagen) according to the manufacturer's protocol. Concentrations of cfDNA were quantified with the Qubit® 3.0 Fluorometer (Thermo Fisher Scientific), fragment sizes were analyzed with the 4200 TapeStation (Agilent Technologies). A panel of 35 highly polymorphic SNP (single‐nucleotide polymorphism)‐assays^28^ was used to discriminate rd‐cfDNA from dd‐cfDNA.

### 2.3 Target‐specific preamplification and cfDNA analysis

Target preamplification of cfDNA was performed using pooled primers from the 35 SNP panels. The preamplified cfDNA was quantified by ddPCR using specific single SNP assays from the SNP panel, based on the difference in genotypes between the recipient and its donor. Multiple SNP assays were used for each patient, all experiments included no template controls. The copies generated by droplet ddPCR for each allele at each SNP locus were calculated using Quanta Soft (Bio‐Rad). The mean value from triplicate assays was used to calculate the absolute levels of dd‐cfDNA, rd‐cfDNA, and DF. DF was defined as the percentage of dd‐cfDNA of the total amount of cfDNA.

The dd‐cfDNA, rd‐cfDNA, and DF levels were compared with clinical events focusing on the relationship between elevated DF and CLAD.

### 2.4 Determination of assay performances

The efficiency of target‐specific preamplification was determined using a cfDNA control, from normal donor plasma, in the range of 0.5–32 ng. cfDNA was preamplified in a single multiplex reaction for 10 cycles and monitored for individual SNP assays by real‐time PCR and SYBR‐green in triplicates. The quantitative real‐time PCR (qPCR) profiles for the SNP assays used in this study are shown in Figure S1.

Limit of blank (LOB), limit of detection (LOD), and limit of quantification (LOQ) were defined based on guidelines in Reference [29]. Reference materials were developed by mixing DNA with known genotypes in separate sample panels. Genomic DNA (WTa) (50 and 100 ng), homozygous for a defined SNP, was mixed with trace amounts of genomic DNA (WTb) homozygous for the reciprocal SNP allele, at target levels ranging from 0.005% to 1% to simulate different amounts of DNA originating from the donor. The samples were analyzed in triplicates by ddPCR and the results are shown in Table S1.

The LOB was set as the highest level of donor DNA that might be found when replicates of blank samples (no donor genome present) are tested: LOB = mean (blank) + 1.645 × SD (blank). The LOD was set at the lowest copy number concentration that could be distinguished from LOB with >95% certainty: LOD = LOB + 1.645 × SD (low‐concentration sample). The LOQ was set as the lowest analyte concentration for which the method provides results with an acceptable uncertainty.

### 2.5 Graphs and statistics

All graphs were made using GraphPad® Prism 9.0.2. Values were shown as median with standard deviation. Comparisons at the group level were performed using the Mann–Whitney *U* test. *p* < .05 was considered statistically significant.

## 3 RESULTS

### 3.1 Assay performances

The efficiency of preamplification was determined using a cfDNA standard and qPCR to monitor individual SNP assays. The qPCR profiles for the SNP assays are seen in Figure S1. No changes in allelic distribution for the SNP assays could be detected within the range of cfDNA concentrations.

LOB was empirically determined as the 95th percentile of 44 blank samples to equal 0.016% dd‐DNA. The median % dd‐DNA value for blank samples was 0.004%, see Table S1. The LOD value was calculated to equal 0.055% dd‐DNA. The LOQ value was determined to be equal to LOD, corresponding to >13 times above the median of the blank. The *R* ^2^ values determined for each dilution series of the respective assay were >0.99 in all cases indicating that the assays were sufficiently accurate to allow LOQ = LOD (Table S1).

**Figure 1:**
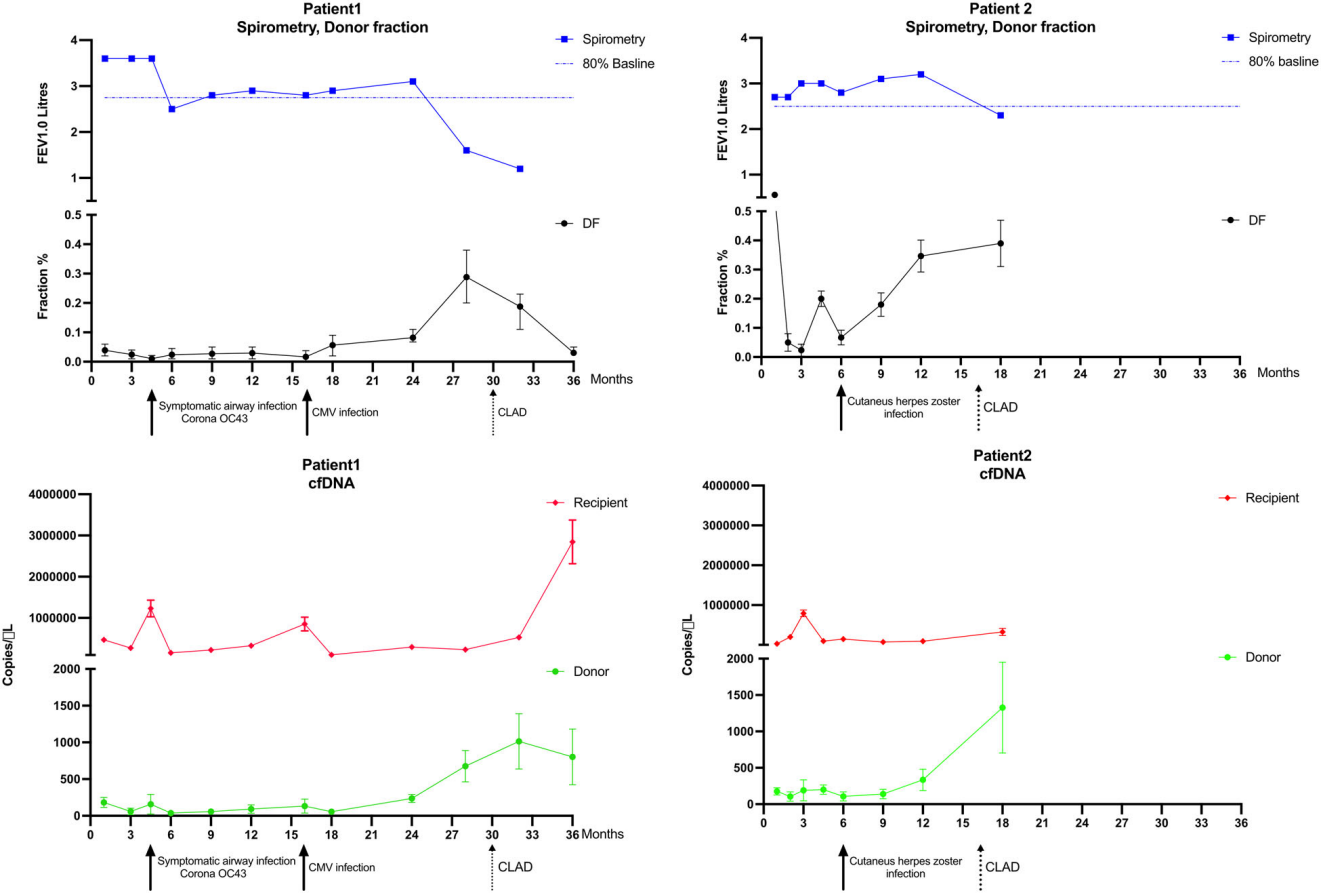
Kinetics of spirometry and cell‐free DNA (cfDNA) levels in serum for Patients 1 and 2 during follow‐up. Solid arrows mark clinical events; dotted arrows mark chronic lung allograft dysfunction (CLAD). The blue line represents forced expiratory volume during the first second (FEV1). The horizontal dotted blue line in the same graph represents 80% of the baseline value, that is, the threshold value for CLAD. The black line represents the donor fraction (DF) of cfDNA. The red line represents copies of cfDNA from the recipient and the green line represents copies of cfDNA from the donor.

**Figure 2:**
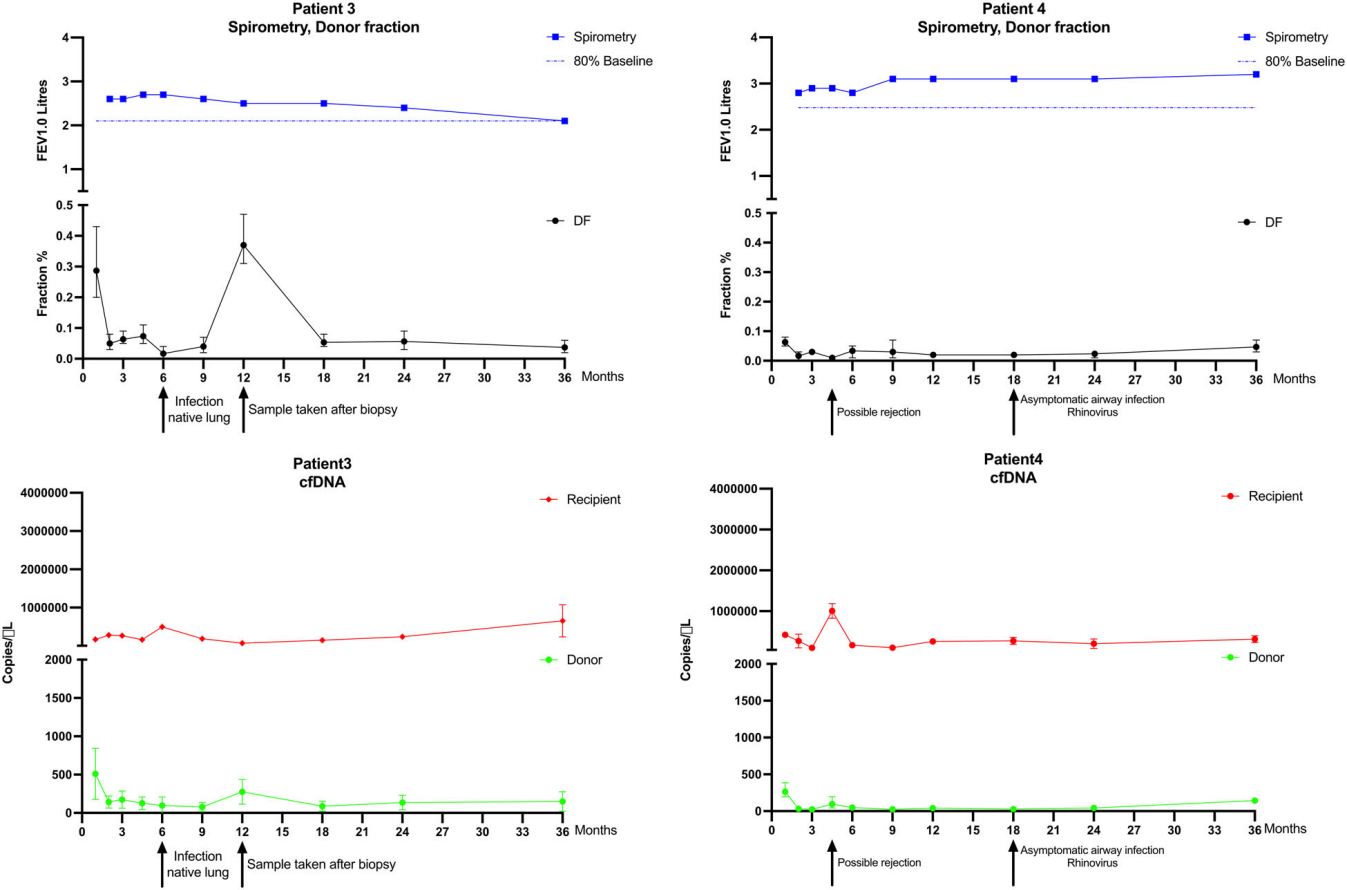
Kinetics of spirometry and cell‐free DNA (cfDNA) levels in serum for Patients 3 and 4 during follow‐up. Solid arrows mark clinical events; dotted arrows mark chronic lung allograft dysfunction (CLAD). The blue line represents forced expiratory volume during the first second (FEV1). The horizontal dotted blue line in the same graph represents 80% of the baseline value, that is, the threshold value for CLAD. The black line represents the donor fraction (DF) of cfDNA. The red line represents copies of cfDNA from the recipient, and the green line represents copies of cfDNA from the donor.

A total of 42 samples were analyzed from the four transplant recipients. Between 3 and 5 informative SNP assays were used for each patient. Figures 1 and 2 display levels of dd‐cfDNA and rd‐cfDNA (copies/µl) over time, along with spirometry data to reveal CLAD development. DF values varied from low levels <0.1% in samples with no clinical events to 0.3%–0.4% in samples associated with verified CLAD.

Baseline clinical data for the four patients are presented in Table 1. All recorded clinical events are described in Table 1. Patients 1 and 2 developed CLAD within the designated follow‐up period of 36 months, while Patients 3 and 4 did not.

#### 3.1.1 Patient 1

For Patient 1, two events were recorded with corresponding peaks of dd‐cfDNA and rd‐cfDNA (Figure 1). The first event represented a symptomatic upper respiratory tract infection with the common cold coronavirus OC43. The second event represented cytomegalovirus (CMV) reactivation with elevated CMV DNA levels in serum. Treatment with valgancivlovir was given for 2 weeks, and CMV levels reverted. Levels of dd‐cfDNA and rd‐cfDNA were simultaneously elevated, no elevation of DF was seen at any of the two recorded clinical events. However, dd‐cfDNA levels increased at 24 months resulting in an elevated DF. At 30 months, while the dd‐cfDNA levels were still elevated, the patient was diagnosed with CLAD, became more immobilized, and suffered weight loss, noted at the extra visit at 34 months. The weight loss and malaise were assumed to be associated with the CLAD diagnosis. Levels of rd‐cfDNA gradually continued to rise after 34 months, while dd‐cfDNA remained elevated. This results in a decreasing DF with persisting CLAD diagnosis. The patient was unable to perform spirometry at the last recorded visit due to fatigue.

#### 3.1.2 Patient 2

For Patient 2, only one clinical event was recorded (Figure 1), represented by a varicella‐zoster virus (VZV) reactivation with cutaneous shingles, treated with oral acyclovir for 7 days. No change in neither rd‐cfDNA nor dd‐cfDNA was detected during this event. A peak in rd‐cfDNA at Month 3 had no clinical correlate. A slight elevation of the dd‐cfDNA was seen from Month 6 and onwards, without change of rd‐cfDNA, resulting in a marked elevation of DF. CLAD diagnosis was made at 14 months. The patient deteriorated and eventually died at 38 months. The patient was unable to adhere to follow‐up visits after 18 months.

#### 3.1.3 Patient 3

For Patient 3, a single‐lung transplant, two clinical events were recorded (Figure 2). At 6 months, the patient experienced lower respiratory tract infection with x‐ray opacities in the native lung, treated with antibiotics and prednisone, with a corresponding elevation of rd‐cfDNA levels. At 12 months, there was an elevation of dd‐cfDNA. This sample was, by mistake, obtained directly after the 12‐month protocol bronchoscopy, including bronchial biopsies, instead of just before. The rd‐cfDNA levels started to rise from Month 18 and onwards. The patient developed chronic kidney disease of stage 3b at 12 months which deteriorated to stage 5 during follow‐up.

#### 3.1.4 Patient 4

For Patient 4, two clinical events were recorded (Figure 2). At 4.5 months, the patient experienced an episode of shortness of breath and elevated C‐reactive protein without an etiologic diagnosis, treated with a short tapering dose of corticosteroids. There was an elevation of both dd‐cfDNA and rd‐cfDNA however, the increase in rd‐cfDNA was much more pronounced, which resulted in no elevation of the DF. At 18 months, rhinovirus was detected in surveillance nasopharyngeal testing in the absence of symptoms. At this point no effect is noted on cfDNA. There are no events or trends during the remainder of the follow‐up and the cfDNA also remains stable. The patient eventually developed a slowly progressing CLAD more than 3 years after the designated follow‐up period.

### 3.2 Overall performance

For both patients with CLAD, an elevated quota of dd‐cfDNA and rd‐cfDNA in the form of DF could be detected before the clinical diagnosis was set. In all events where clinical significance would be expected, this was reflected in cfDNA levels depending on whether the donor organ or recipient body was mainly affected. Only one episode of cfDNA elevation without a clear clinical correlate was detected. All severe infections were found to have a correlation with cfDNA levels.

**Figure 3:**
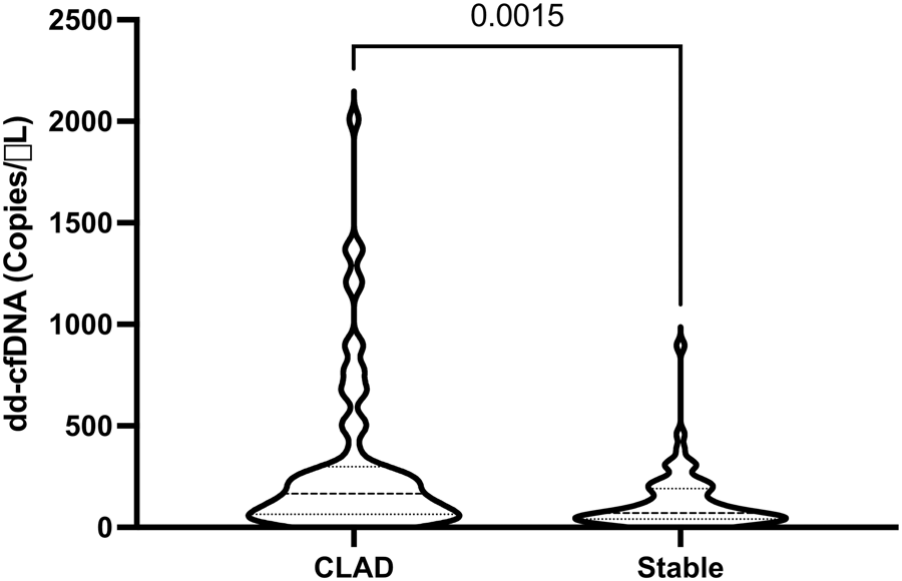
Cumulative pooled levels of donor‐derived cell‐free DNA (dd‐cfDNA) visualized. Patients 1 and 2 are denominated chronic lung allograft dysfunction (CLAD), and Patients 3 and 4 are denominated as stable.

The mean levels of dd‐cfDNA of all samples collected within the entire follow‐up period of the first 36 months were higher (*p* = .0015) among patients who developed CLAD within the follow‐up period (Figure 3).

## 4 DISCUSSION

We propose a novel method for detecting and quantifying both dd‐cfDNA and rd‐cfDNA that can be performed using frozen serum samples at a low cost and a rapid analysis turnover. The levels of the different origins of cfDNA are associated with clinical events where the elevation of dd‐cfDNA mirrors damage to the graft. Raised levels of rd‐cfDNA correlated with several infection episodes of the host. We recorded a doubling of the levels from an individual baseline associated with a recorded clinical event in close proximity in all but two cases. A consistently elevated DF was preceding CLAD diagnosis in both patients and noted up to 6 months before CLAD was evident.

The events with observational proximity in time, between event and cfDNA elevation, are all of a more severe nature. The three events where both rd‐cfDNA and dd‐cfDNA were elevated represent infectious complications. Most interestingly, one of those is a symptomatic common cold coronavirus respiratory tract infection, previously suggested to be associated with long‐term CLAD development.^8^ The second event was a CMV reactivation and the third event was an unclear episode of deterioration without elevation of inflammatory markers (Patient 4) and negative bronchoscopy findings of all modalities where no etiology could be established. This patient recovered swiftly after corticosteroid treatment.

There were two recorded clinical events without any marked elevation of cfDNA from either donor or recipient. These cases were an asymptomatic rhinovirus infection and a case of VZV reactivation with shingles, indicating that these two events represent less severe complications. Quantification of cfDNA could thus possibly be used to determine the clinical importance of infectious events in conjunction with other tests. This could possibly differ severe infectious events from those of limited importance at an earlier stage.

In‐detail interpretation of the results from each of the patients is warranted. For Patient 1 (Figure 1), there is a marked increase of dd‐cfDNA from Month 24 and onwards. The increase in DF seen at 18 months, is due to the concomitant decline in rd‐cfDNA, showing the importance of separately investigating dd‐ and rd‐cfDNA and not only DF. Although this patient's condition deteriorated, the DF, which was initially elevated, normalized which suggests an issue when looking at only DF for detecting CLAD and suggests that the separate analysis of dd‐ and rd‐cfDNA may improve the interpretation of DF levels. Patient 2 (Figure 1) had an elevation of dd‐cfDNA from 12 months without any simultaneous alteration of rd‐cfDNA. There was no competing event besides CLAD. The DF was already elevated 6 months before the diagnosis. In this case, the elevation of DF could be observed as an early warning sign of CLAD development. One episode of transient elevation of rd‐cfDNA in Patient 2 showed no relation to any clinical event and the reason could not be determined. Under‐reporting of symptoms, lack of adherence to follow‐up, and DF elevation for hitherto unknown reasons are possible causes. Patient 3 (Figure 2) showed elevated rd‐cfDNA levels from Month 18 and onwards. The only recognized ongoing event was progressive renal failure. The cfDNA is likely to be eliminated by the kidneys but to what extent is currently not known.^30^ However, the patient is a single lung transplant recipient and thus had a remaining lung afflicted with idiopathic pulmonary fibrosis (IPF). The extent of cfDNA released by IPF progression is unknown. In previous studies, measured cfDNA levels for single LTx have been doubled on organ mass assumption.^20^ Only unmodified values are used as the levels are related to a relative increase in the current study. One episode of isolated transient dd‐cfDNA elevation was seen directly following a protocol biopsy of the transplanted lung. The biopsy was performed a few hours before the blood sample was collected, contrary to the plan, and the cfDNA elevation could be explained by minor damage to the transplanted organ secondary to the biopsy procedure.^31^ This observation also emphasizes the importance of sequential testing as singular tests can indicate organ damage but not the reason behind it. Patient 4 (Figure 2) has a transient elevation of rd‐cfDNA at four and a half months but shows no persistent cfDNA levels during the follow‐up. The event at four and a half months could have been an acute rejection. However, the dd‐cfDNA elevation was relatively moderate compared to the rd‐cfDNA. The cause of the acute deterioration could not be determined with certainty. CLAD in Patient 4, which developed 3 years after the last sample, is unlikely to be detected, given the extended period of time until the onset of symptoms.

The average amounts of dd‐cfDNA were higher in the two patients that eventually developed CLAD during the sample period (Figure 3). This is in line with previous findings.^32^

de Vlaminck et al.^33^ had previously presented evidence of a correlation between DF and graft injury in 51 prospectively followed patients.^33^ These findings support the usefulness of dd‐cfDNA as a biomarker after lung transplantation. Moreover, Agbor‐Enoh et al.^20^ has previously published a retrospective study where an association between level of DF and both allograft and overall survival was presented. This study suggests an association between an isolated elevation of DF and graft damage. Both studies assumed mass correlation for single lung transplanted patients by doubling the dd‐cfDNA, while no such assumption was made in the present study. Neither study reports results on an individual level, why the accordance of clinical events and cfDNA levels cannot be compared.

Notably, in Agbor‐Enoch et al.'s study,^20^ elevated DF was defined as ≥1%. In the current report, DF levels are between 0.2% and 0.4% when associated with CLAD. The difference is most likely due to higher levels of recipient genomic DNA in the samples in the current data set,^34^ which, in turn, is caused by preanalytical factors such as degree of hemolysis in collection tubes, transport times, and centrifugation procedures. However, the diverging methodologies preclude any direct comparisons of rd‐cfDNA levels.

All of the samples were stored frozen for more than 5 years in the present study. The study was designed to detect a vast majority of infectious events and combined surveillance testing with testing in acute events. Thus, the samples are very well suited to investigate the viability of the current method as a biomarker. The high temporal accordance between cfDNA and clinical events implies that the method might be possible to use in a clinical setting. It also shows the advantage of having dd‐cfDNA and rd‐cfDNA levels measured as well when interpreting DF. The results also confirm the capacity to analyze previously frozen sera. The possibility of freezing and storing samples opens up for sampling at a secondary site which means that the rather complicated method only needs to be set up in a limited number of laboratories to cover one or several transplant programs.

The study analyzed a limited number of patients. This and the retrospective design leads to limited direct clinical usefulness of the results. The samples were not collected and prepared according to the currently used protocol.^21^ Therefore, we cannot draw clear conclusions on causal relationships between observed clinical events and cfDNA alterations. The numbers as well preclude relevant statistical calculations. The original study was performed some years ago, and follow‐up routines have changed, for example, the number of total lung capacity measurements performed during follow‐up and the number of biopsies taken has changed over time.

Future studies of the current method for cfDNA analysis in lung transplant patients need to be in larger cohorts to define inter‐patient variability better. The studies should include analyses of cfDNA response to different non‐CLAD types of allograft injury, such as infections, acute rejection, and biopsies, as demonstrated in the current study. Also, cfDNA response to different types of CLAD as well as post‐CLAD dynamics of cfDNA needs to be analyzed. Furthermore, prospective sampling in parallel with current monitoring practices, for example, donor‐derived antibodies, would be of great interest.

In conclusion, the present pilot study shows that analysis of the quantity and relative proportion of donor‐ and recipient‐derived cfDNA using this novel method is feasible and that circulating levels may reflect important clinical events like allograft damage and significant infectious complications following lung transplantation. The results hint at the possibility of the method being used to detect CLAD. Further prospective research is warranted to validate the measurement of cfDNA to predict complications in a clinical setting.

## AUTHOR CONTRIBUTIONS

Jesper M. Magnusson conceptualized the idea, compiled data, interpreted data, drafted the manuscript. Anne Ricksten was pivotal to the methodology, supervised the laboratory analyses, supervised data accuracy, interpreted data, and contributed to the writing of the manuscript. Göran Dellgren supervised the study and contributed to the writing of the manuscript. Carina Wasslavik performed the laboratory analyses and contributed to the writing of the manuscript. Rickard Nordén refined the idea, interpreted data, and contributed to the writing of the manuscript. Johan Westin refined the idea, managed overall performance of the study, interpreted data, and contributed to writing the manuscript. Jens Boehmer conceptualized the idea, Interpreted the data, and contributed to writing the manuscript.

## Supporting information

Supplementary information.

Supplementary information.

## Data Availability

Data are available on request due to privacy/ethical restrictions.
