# Supplementary material for: Cell‐free DNA as a biomarker after lung transplantation: A proof‐of‐concept study
Source: Immun Inflamm Dis. 2022 Apr 19;10(5):e620. doi: 10.1002/iid3.620 (PMC9017613; doi:10.1002/iid3.620)
Supplement: Supplementary file 1 — Supplementary information. [file IID3-10-e620-s002.docx]

Supplemental Figure 1: Target-specific preamplification was performed on a cfDNA

control in the range of 0.5 to 32 ng per reaction, following monitoring of individual SNP

assays by real-time PCR and SYBR-green in triplicates. The qPCR profiles for the

preamplified (PA) SNP assays # 2, 8, 16, 17, 21, 23, 26, 32, 33, 36 and 37 are shown in the

figure. Cq = quantification cycle.
