# Supplementary material for: Cell‐free DNA as a biomarker after lung transplantation: A proof‐of‐concept study
Source: Immun Inflamm Dis. 2022 Apr 19;10(5):e620. doi: 10.1002/iid3.620 (PMC9017613; doi:10.1002/iid3.620)
Supplement: Supplementary file 2 — Supplementary information. [file IID3-10-e620-s001.docx]

| **Assay** | **Total DNA/assay ng** | **1% donor-DNA** | **0.5% donor-DNA** | **0.1% donor-DNA** | **0.05% donor DNA** | **0.01% donor-DNA** | **0.005% donor-DNA** | **WT a 0% donor-DNA** | **WT b 0% donor-DNA** | **R^2^** |
| --- | --- | --- | --- | --- | --- | --- | --- | --- | --- | --- |
| #2 | 50 | 1.1 | 0.61 | 0.13 | 0.04 | 0.003 | 0.000 | 0.000 | 0.000 | 0.9982 |
| #2 | 100 | 1.1 | 0.56 | 0.12 | 0.06 | 0.007 | 0.002 | 0.000 | 0.000 | 0.9998 |
| #8 | 50 | 1.1 | 0.63 | 0.14 | 0.14 | 0.057 | 0.067 | 0.001 | 0.001 | 0.9965 |
| #8 | 100 | 1.0 | 0.51 | 0.13 | 0.11 | 0.064 | 0.039 | 0.001 | 0.000 | 0.9986 |
| #16 | 50 | 1.1 | 0.55 | 0.12 | 0.06 | 0.016 | 0.012 | 0.000 | 0.000 | 0.9999 |
| #16 | 100 | 1.1 | 0.55 | 0.11 | 0.07 | 0.008 | 0.002 | 0.000 | 0.000 | 0.9992 |
| #17 | 50 | 1.0 | 0.45 | 0.12 | 0.06 | 0.006 | 0.006 | 0.000 | 0.000 | 0.9973 |
| #17 | 100 | 1.0 | 0.53 | 0.12 | 0.03 | 0.008 | nd | 0.000 | 0.000 | 0.9988 |
| #21 | 50 | 1.0 | 0.47 | 0.14 | 0.04 | 0.006 | 0.006 | 0.000 | 0.000 | 0.9957 |
| #21 | 100 | 1.2 | 0.49 | 0.10 | 0.06 | 0.010 | 0.011 | 0.000 | 0.000 | 0.9917 |
| #23 | 50 | 1.0 | 0.56 | 0.16 | 0.12 | 0.028 | 0.015 | 0.000 | 0.000 | 0.9961 |
| #23 | 100 | 0.9 | 0.53 | 0.11 | 0.08 | 0.019 | 0.009 | 0.000 | 0.000 | 0.9964 |
| #26 | 50 | 0.9 | 0.47 | 0.11 | 0.11 | 0.048 | 0.081 | 0.002 | 0.001 | 0.9970 |
| #26 | 100 | 1.0 | 0.50 | 0.15 | 0.10 | 0.062 | 0.040 | 0.001 | 0.000 | 0.9990 |
| #32 | 50 | 1.0 | 0.53 | 0.13 | 0.08 | 0.049 | 0.034 | 0.000 | 0.035 | 0.9999 |
| #32 | 100 | 0.9 | 0.49 | 0.10 | 0.05 | 0.040 | 0.025 | 0.000 | 0.018 | 0.9990 |
| #33 | 50 | 0.9 | 0.47 | 0.10 | 0.05 | 0.012 | 0.011 | 0.000 | 0.000 | 1.0000 |
| #33 | 100 | 0.9 | 0.47 | 0.09 | 0.04 | 0.006 | 0.004 | 0,000 | 0.000 | 0.9992 |
| #36 | 50 | 1.1 | 0.62 | 0.16 | 0.09 | 0.078 | 0.093 | 0.000 | 0.000 | 0.9975 |
| #36 | 100 | 1.1 | 0.58 | 0.19 | 0.11 | 0.074 | 0.053 | 0.000 | 0.001 | 0.9991 |
| #37 | 50 | 1.1 | 0.48 | 0.11 | 0.07 | 0.039 | 0.034 | 0.040 | 0.040 | 0.9958 |
| #37 | 100 | 0.9 | 0.51 | 0.13 | 0.07 | 0.032 | 0.018 | 0.000 | 0.030 | 0.9974 |
| #2 | 50 | 1.1 | 0.61 | 0.13 | 0.04 | 0.003 | 0.000 | 0.000 | 0.000 | 0.9982 |
| #2 | 100 | 1.1 | 0.56 | 0.12 | 0.06 | 0.007 | 0.002 | 0.000 | 0.000 | 0.9998 |
| #8 | 50 | 1.1 | 0.63 | 0.14 | 0.14 | 0.057 | 0.067 | 0.001 | 0.001 | 0.9965 |
| #8 | 100 | 1.0 | 0.51 | 0.13 | 0.11 | 0.064 | 0.039 | 0.001 | 0.000 | 0.9986 |

Supplemental table 1: dd-DNA assay limit of blank (LOB) and limit of detection (LOD).

Supplemental, Table 1. Mean % dd-DNA for low levels of donor DNA (WTb) in a background of 50 and 100ng, respectively, genomic DNA (WTa) are shown for the SNP assays 2,8,16,17,21,23,26,32,33,36 and 37. LOB= 0.016% was determined as the 95^th^ percentile of % donor DNA for 44 blank samples. The LOD value was calculated to equal 0.055% dd-DNA. The R-square (R2) values were determined for each dilution series of respective assay.
